# Mutational Characterization of Cutaneous Melanoma Supports Divergent Pathways Model for Melanoma Development

**DOI:** 10.3390/cancers13205219

**Published:** 2021-10-18

**Authors:** David Millán-Esteban, María Peña-Chilet, Zaida García-Casado, Esperanza Manrique-Silva, Celia Requena, José Bañuls, Jose Antonio López-Guerrero, Aranzazu Rodríguez-Hernández, Víctor Traves, Joaquín Dopazo, Amaya Virós, Rajiv Kumar, Eduardo Nagore

**Affiliations:** 1 School of Medicine, Universidad Católica de València San Vicente Mártir, 46001 Valencia, Spain; david.millan@ucv.es; 2 Laboratory of Molecular Biology, Fundación Instituto Valenciano de Oncología, 46009 Valencia, Spain; zgarcia@fivo.org (Z.G.-C.); jalopez@fivo.org (J.A.L.-G.); 3 Clinical Bioinformatics Area, Fundación Progreso y Salud, Hospital Virgen del Rocío, 41013 Sevilla, Spain; maria.pena.chilet.ext@juntadeandalucia.es (M.P.-C.); joaquin.dopazo@juntadeandalucia.es (J.D.); 4 Bioinformatics in Rare Diseases (BiER), Centro de Investigación Biomédica en Red de Enfermedades Raras (CIBERER), 41013 Sevilla, Spain; 5 Computational Systems Medicine, Institute of Biomedicine of Seville (IBIS), Hospital Virgen del Rocío, 41013 Sevilla, Spain; crequena@fivo.org; 6 Department of Dermatology, Fundación Instituto Valenciano de Oncología, 46009 Valencia, Spain; emanrique@fivo.org (E.M.-S.); arodriguezh@fivo.org (A.R.-H.); 7 Department of Dermatology, El Instituto de Investigación Sanitaria y Biomédica de Alicante (ISABIAL), Hospital General Universitario de Alicante, 03010 Alicante, Spain; banuls_jos@gva.es; 8 Department of Pathological Anatomy, Fundación Instituto Valenciano de Oncología, 46009 Valencia, Spain; vtraves@fivo.org; 9 Fundación Progreso y Salud-ELIXIR-es, Hospital Virgen del Rocío, 41013 Sevilla, Spain; 10 Skin Cancer and Aging Lab, Cancer Research UK Manchester Institute, University of Manchester, Manchester SK10 4TG, UK; amaya.viros@cruk.manchester.ac.uk; 11 Division of Functional Genome Analysis, Deutsches Krebsforschüngzentrum, 69120 Heidelberg, Germany; R.Kumar@Dkfz-Heidelberg.de; 12 Department of Molecular Biology of Cancer, Institute of Experimental Medicine of the Czech Academy of Sciences, Videnska, 142 20 Prague, Czech Republic; 13 Institute of Medical Biometry and Informatics, University of Heidelberg, 69117 Heidelberg, Germany

**Keywords:** melanoma, etiopathogeny, mutations

## Abstract

**Simple Summary:**

The divergent pathway model established at least two approaches for melanoma development. One was related to a propensity to melanocytic proliferation (nevogenic), and the other was associated with an accumulation of solar damage (CSD). We conducted a retrospective study to examine whether this model had a molecular support using sequencing and bioinformatic tools on a set of cutaneous melanomas corresponding to these two groups. We found that the nevogenic melanomas were associated with mutations in *BRAF*, while the CSD melanomas were associated with mutations in *NF1*, *ROS1*, *GNA11*, and *RAC1*. We concluded that nevogenic and CSD melanomas constitute two different biological entities.

**Abstract:**

According to the divergent pathway model, cutaneous melanoma comprises a nevogenic group with a propensity to melanocyte proliferation and another one associated with cumulative solar damage (CSD). While characterized clinically and epidemiologically, the differences in the molecular profiles between the groups have remained primarily uninvestigated. This study has used a custom gene panel and bioinformatics tools to investigate the potential molecular differences in a thoroughly characterized cohort of 119 melanoma patients belonging to nevogenic and CSD groups. We found that the nevogenic melanomas had a restricted set of mutations, with the prominently mutated gene being *BRAF*. The CSD melanomas, in contrast, showed mutations in a diverse group of genes that included *NF1*, *ROS1*, *GNA11*, and *RAC1*. We thus provide evidence that nevogenic and CSD melanomas constitute different biological entities and highlight the need to explore new targeted therapies.

## 1. Introduction

The divergent pathways model suggested the clinical classification of cutaneous melanoma into two groups: one associated with melanocyte proliferation proneness (nevogenic), and the other with cumulative solar damage (CSD) [1]. Both groups share an initiation step in which the activation of melanocytes proceeds via exposure to ultraviolet radiation (UVR) early in life and host factors. Afterward, the progression towards melanoma diverges depending on exogenous and endogenous factors. The nevogenic melanomas arise in individuals constitutively predisposed to melanocytic proliferation, characterized by a high nevi count, with little involvement of acquired UVR damage. Those tumors appear in young/middle-aged people on intermittently sun-exposed areas, such as the trunk.

In contrast, the CSD melanomas occur mainly in individuals with a low number of nevi, located on chronically sun-exposed skin, such as the head and neck, with solar elastosis on the healthy skin surrounding the melanoma. Those tumors emerge after a lifetime of cumulative sun exposure in older patients [2,3]. Epidemiological studies have confirmed the divergent pathways hypothesis based on the distribution and number of nevi, UV-related skin damage, patient age at diagnosis, and other clinical aspects [4,5,6,7]. Furthermore, these two populations would correspond to subgroups within the current WHO classification, which differentiates between high-CSD and low-CSD melanomas but considers, for the latter, the proneness to melanocytic proliferation [8].

The differential molecular characterization of tumors from two etiopathogenic pathways, despite the advanced sequencing initiative, has remained uninvestigated even two decades later and has demonstrated clinical relevance [9,10]. The sequencing studies on cutaneous melanoma, in general, showed that the most prevalent mutations include those in *BRAF*, *TERT* promoter (*TERTp*), *NRAS*, *NF1*, *ARID2*, and *TP53*. Based on the mutational pattern, cutaneous melanoma is classified into four molecular mutually exclusive subtypes. The four groups are based on mutations in *BRAF* (“BRAF+”), *NRAS/HRAS/KRAS* (“RAS+”), *NF1* (“NF1+”), or the absence of those three types of mutations, referred to as triple wild types (“3wt”) [11,12,13].

The big genomic data repositories can foster models to predict the relevant aspects of molecular and patient phenotypes. Such models, based on the molecular pathways, reveal relevant features of the disease. These novel tools allow prediction about the effects of alterations in the modelled system in silico, with potential new therapeutic targets and to predict the functional impact of loss-of-function (LoF) mutations on the different cell mechanisms in complex diseases [14,15,16].

This study sequenced tumors from cutaneous melanoma patients developed through two mutually exclusive routes to understand the molecular differences and similarities using a custom gene panel covering most frequently altered genes. The data were analyzed using comprehensive bioinformatics tools to characterize two seemingly different types of melanoma.

## 2. Results

### 2.1. Mutational Distribution among Nevogenic and CSD Melanomas

A total of 119 primary melanomas provided informative sequences: 82 (68.9%) from the nevogenic group and 37 (31.1%) from the CSD group (Figure S1). The median age of the patients at diagnosis was 59 years, and they included 65 men (54.6%) and 54 women (45.4%). The nevogenic group included 42 (51.2%) men and 40 (48.8%) women, whereas the CSD group included 23 (62.2%) men and 14 (37.8%) women. A detailed description of the demographic and clinicopathological characteristics of the cohort is displayed in Table 1.

Overall, the most mutated genes/loci were *TERTp* (52.2%), *BRAF* (50.4%), *NF1* (16.8%), *NRAS* (13.4%), *ROS1* (11.8%), and *TP53* (10.9%), with the remaining genes investigated having a mutational frequency of <10% (Table 2). Of the total, 106 (89.1%) melanomas were classified into the four major groups: 48/119 (40.3%) “*BRAF+*”, 15/119 (12.6%) “*RAS+*”, 10/119 (8.4%) “*NF1+*”, and 33/119 (27.7%) “3wt”; however, 13 (10.9%) patients, due to mutations in overlapping genes, eluded classification: 3/119 (2.5%) melanomas showed both a *BRAF* and *RAS* mutation, 9/119 (7.6%) showed both a *BRAF* and *NF1* mutation, and 1/119 (0.8%) showed a mutation in both *RAS* and *NF1* (Table 2). A graphical representation of the mutational concurrence can be found in Figure S2.

**Figure 1:**
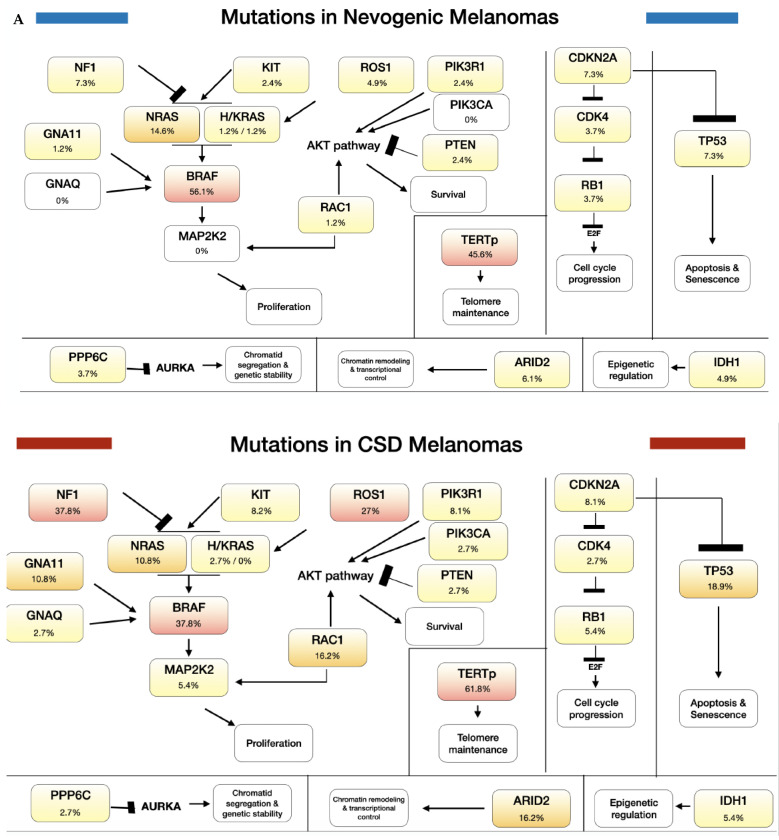

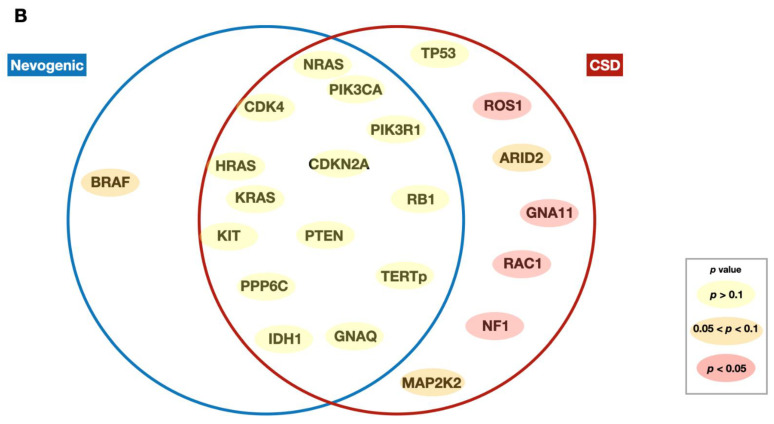
Mutational prevalence and mutational association for etiopathogenic pathways. Mutational distribution between nevogenic and CSD melanomas. Frequency of mutations in the different genes for the nevogenic and CSD groups (**A**); Graphical representation of the association of mutations in the different genes with either group based on their *p*-value (**B**).

The nevogenic tumors had a higher frequency of *BRAF* mutations than the CSD melanomas, although the difference was not statistically significant (46/82, 56.1% vs. 14/37, 37.8%; *p* = 0.077). In contrast, the CSD melanomas had a higher frequency of mutations than the nevogenic melanomas in *NF1* (14/37, 37.8% vs. 6/82, 7.3%; *p* < 0.001), *ROS1* (10/37, 27.0% vs. 4/82, 4.9%; *p* = 0.001), *GNA11* (4/37, 10.8% vs. 1/82, 1.2%; *p* = 0.032), and *RAC1* (6/37, 16.2% vs. 1/82, 1.2%; *p* = 0.004; Table 3; Figure 1; Figure S3). The differences were further assessed by univariate logistic regression, and, after adjustment, only the mutations in *NF1* and *ROS1* remained independently associated with the CSD melanomas (Figure S4).

The mean comparison showed a statistically significant difference in the total number of pathogenic mutations between the nevogenic and CSD melanomas (1.9 vs. 3.4; *p* = 0.029), but no differences were found in the number of UV-induced mutations.

The distribution of the molecular subtypes among the described major etiopathogenic groups was the following: the “*BRAF+*” subtype was significantly associated with the nevogenic melanomas (39/75, 52.0% vs. 9/31, 29.0%), while “*NF1+*” was related to the CSD melanomas (1/37, 1.3% vs. 9/31, 29.0%) (*p* < 0.001) (Table 3).

### 2.2. Mechanistic Analysis of Pathways

**Figure 2:**
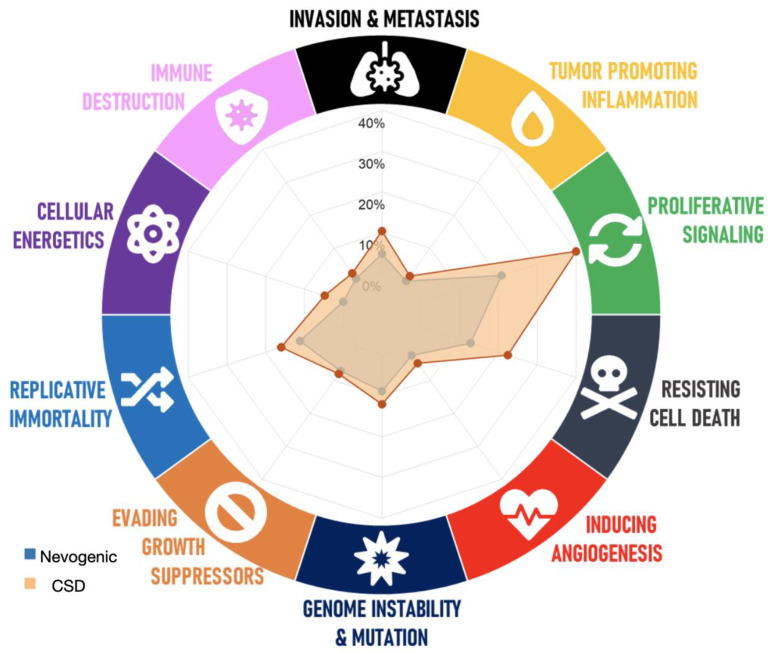
Radar plot of cancer hallmarks. This graphical representation shows the distribution of dysregulated circuits for each group. Percentages are used as an approximation to reflect the differences in the overall number of dysregulated circuits per hallmark found in each group.

The mechanistic analysis based on the mutational profiles to predict the effect on normal skin showed that 67 circuits were significantly dysregulated in the nevogenic melanomas (66 upregulated; 1 downregulated), and 122 circuits were dysregulated in the CSD melanomas (109 upregulated; 13 downregulated). Fifty-one circuits were statistically significantly higher in the CSD than in nevogenic melanomas (Figure S5). A radar plot was visualized with the altered pathways in the context of the annotated hallmarks of cancer (Figure 2). The plot showed that the mutational profiles from the CSD melanomas had a higher number of dysregulated circuits (counts; “Cs”) annotated to hallmarks of cancer than those from the nevogenic melanomas, especially when considering proliferative signaling (26 vs. 50 Cs), replicative immortality (14 vs. 20 Cs), resisting cell death (16 vs. 28 Cs), and genome instability and mutation (11 vs. 15 Cs). The enrichment analysis based on simulated circuit activity data from normal skin showed that dysregulations in proliferative signaling and replicative immortality were statistically significant in the nevogenic (*p* = 0.01; *p* = 0.002) and CSD (*p* = 0.0004; *p* = 0.0002) melanomas compared to their corresponding normal skin (Figure S6).

## 3. Discussion

Cutaneous melanoma is a complex disease sorted by different characteristics. However, a clinical classification represents a helpful approach given the disease’s etiology, evolution, and mutational status. The divergent pathways model confirmed through clinical and epidemiological studies posits two different cutaneous melanoma groups (nevogenic and CSD). In this study, based on the molecular characterization of the two divergent groups, we show a higher frequency of mutations in different genes in the CSD melanomas than in the nevogenic melanomas except for *BRAF* mutations.

Although UV-radiation is crucial to the initiation in both melanomas, the CSD type, predicated on chronic sun exposure leading to the accumulation of mutations, reflects the etiology through a typical corresponding mutational signature. The role of UVR on melanocyte proliferation and melanoma development involves direct and indirect mutagenesis processes, including the formation of photoproducts and free radicals resulting from the biochemical interaction of UVA and melanin [17]. Chronic exposure to sun damage leads to multiple alterations affecting the cell’s normal functioning and increases the chance of melanomagenesis. Several prominent genes mutated in CSD melanomas included *NF1*, *ROS1*, *GNA11*, and *RAC1*. *NF1* encodes a GTPase-activating protein that downregulates RAS activity, so loss-of-function mutations activate the MAPK pathway upstream of the RAS. *ROS1*, a receptor tyrosine kinase of the insulin receptor family, is constitutively activated when mutated and also leads to the activation of the pathway; mutated *RAC1* increases the GDP/GTP nucleotide exchange rate, and *GNA11* is a subunit of a G protein-coupled receptor responsible for mediating GTP-binding and limiting the activation of the pathway, and the activating mutations result in the constitutive activation of MAPK [18,19,20,21,22]. Our findings align with the crucial role of the activated MAPK pathway in melanoma for uncontrolled cell proliferation. Based on our in silico simulation analyses using the expression data from normal skin as a reference, the higher number of dysregulated circuits within the proliferation pathway found in CSD melanomas could suggest a more relevant role of this pathway in carcinogenesis than in nevogenic melanomas. Moreover, this dysregulation was more significant in the CSD than in the nevogenic melanomas when compared with their corresponding normal tissue. However, a difference in the number of dysregulated circuits might not translate into actual differences in the individual gene expression levels, so further studies on these two groups should be performed to elucidate whether the proliferation levels are more elevated in the CSD than nevogenic melanomas or not.

Many studies have described that melanomas with a higher tumor mutation burden (TMB) would have a better outcome than those with a lower TMB [23,24]. A higher tumor mutation burden leads to increased potential neoantigens and an improved response to immunotherapy [25,26,27]. Even though our study did not assess TMB, the higher frequency of mutations in the CSD melanomas indicates the trend. Our molecular characterization of the CSD melanomas draws attention to the fact that there are genes specific to this group where mutations have not yet been explored as therapeutic targets. Given the revolution that targeted drugs constituted as inhibitor-based drugs against melanomas harbouring mutations in *BRAF*, *MEK*, and *KIT* [28,29], studies like the present one contribute to the identification of potential lines of work aimed at improving the medical attention of these patients.

Alternatively, the development of melanoma in the absence of accumulated UVR in occasionally exposed anatomical sites remains intriguing, and here we have shown how these nevogenic melanomas were associated with *BRAF* mutations. Multiple studies have shown this association in young nevus-prone patients with melanomas arising at intermittently exposed sites [30,31]. However, this alone does not explain the development of melanoma since *BRAF* mutations have been widely reported in benign melanocytic nevi, which do not necessarily transform into melanoma [32,33,34]. Additional contributing factors are reflected in the literature, with pigment pheomelanin being extensively studied. Compared to eumelanin, pheomelanin has an inherent genotoxic effect via the production of reactive oxygen species (ROS) or consumption of antioxidants, enhancing carcinogenesis independently of UVR [35,36].

Moreover, some studies in rodents suggested additional factors that might contribute to melanomagenesis in similar conditions to nevogenic melanomas. For instance, *BRAF* mutations seem to enhance carcinogenesis resulting from UVB, meaning fewer exposures might be required for melanocyte progression into melanoma [37]. Moreover, previous studies have suggested that the susceptibility to UV might vary through the different sequence regions in the human genome depending on nucleosome structure or bound transcription factors or other factors [38,39]. Hence, a more in-depth sequencing approach covering both coding and non-coding regions could be beneficial to elucidate the real prevalence of UV-signature mutations. Complementarily, germline alterations have not been checked in our study, and they could further explain the development of this group of melanomas, with this missing impetus coming from normal germline variants in RNA-binding proteins or DNA repair genes [40].

The role of TERTp mutations in the development of melanoma has been widely studied since the stabilization of telomeres in cells is one of the hallmarks of cancer [41,42]. In our study, CSD melanomas showed a higher prevalence of mutations within the promoter region of *TERT*, albeit without statistical significance. The lack of association could be due to limited sample size since previous reports had suggested a UVR influence on these mutations, with *TERTp* alterations more frequent in CSD melanomas [43,44]. The *TERT* promoter mutations in previous studies have been shown to associate with markers of poor prognosis, increased tumor growth, hematogenous dissemination, and define the subsets of melanoma patients with poor disease-free and disease-specific survival [45,46,47,48].

Finally, there are some limitations in the present study. The use of a custom gene panel instead of a whole-exome or a whole-genome approach results in some potentially relevant genes being left out (e.g., *MAP2K1*, *CTNNB1*) [49,50]. Moreover, our analysis has focused on SNV and indels and did not include copy number variants, which are relevant as well when characterizing tumors [51].

## 4. Materials and Methods

We designed a retrospective study using the mutational data obtained from next-generation sequencing (NGS) and the information included in our melanoma databases. These contained prospectively collected data from all melanoma patients treated at the Instituto Valenciano de Oncología (IVO) since 2000 and the Hospital General Universitario de Alicante (HGUA) since 1995. Clinical, pathological, and epidemiological data assessed by expert dermatologists and pathologists were included [52]. The study had the approval of the IVO ethics committee.

### 4.1. Patient Selection and Classification

Tumor samples were collected after informed consent and stored as formalin-fixed paraffin-embedded (FFPE) blocks at the corresponding Biobanks after confirmation of melanoma diagnosis by a single pathologist per institution. Patients were classified based on the total number of melanocytic nevi and the histological presence/absence of solar elastosis in the healthy skin surrounding the melanoma. The latter was graded according to a previously described score (11 degrees; range: 0 to 3+) [53]. We selected patients from the two mutually exclusive groups: nevogenic, characterized by the presence of more than 50 nevi and no solar elastosis, and CSD, which included patients with less than 20 melanocytic nevi and moderate to severe solar elastosis.

### 4.2. Sample Preparation

FFPE blocks were retrieved from the corresponding Biobanks, and glass slides were prepared for hematoxylin and eosin staining to guide the macrodissection of the tumor. Either three unstained sections of 10 μm thick tissue were manually scraped, or three 0.6 mm needle biopsies were taken from every sample to ensure a high tumor content, depending on tumor cellularity below or above 70%, respectively.

DNA extraction was performed using the QIAamp^®^ DNA Investigator kit (QIAGEN, Hilden, Germany) with minor modifications. An overnight incubation step at 56 °C for the proteinase K was set to assure complete digestion of the skin, and an optional RNA carrier was added to maximize the extraction yield. Moreover, the NEBNext^®^ FFPE Repair Mix (New England Biolabs, Hertfordshire, UK) was used to repair the DNA, hence minimizing sequencing artifacts due to C:G > T:A changes induced by nucleotide deamination, usually present in FFPE samples. DNA concentration was quantified using the Quant-iT™ PicoGreen™ dsDNA (ThermoFisher, Waltham, MA, USA) fluorimetric assay, and those samples with >2.5 ng/uL continued the process.

### 4.3. Gene Panel and Library Construction

A Custom GeneRead™ DNAseq Targeted Panel V2 (QIAGEN^®^, Hilden, Germany) was designed including coding regions for 21 genes involved in melanomagenesis: *ARID2*, *BRAF*, *CDK4*, *CDKN2A*, *GNA11*, *GNAQ*, *HRAS*, *IDH1*, *KIT*, *KRAS*, *MAP2K2*, *NF1*, *NRAS*, *PIK3CA*, *PIK3R1*, *PPP6C*, *PTEN*, *RAC1*, *RB1*, *ROS1*, and *TP53* (Figure S7). The panel consisted of 633 amplicons distributed in 3 primer pools with an average size of 200 bp (range: 120–275 bp) and an average coverage of 98.2% (range: 75.3–100%). Barcoded libraries were generated from 7.5 ng of DNA per primer pool according to the manufacturer’s instructions, reducing the PCR volume to 12 µL to minimize sample usage. After purification with AMPure beads (Beckam Coulter, Brea, CA, USA), libraries were checked for appropriate size using Genomic DNA ScreenTape in a 4200 TapeStation (Agilent Technologies, Santa Clara, CA, USA). The mutational status of the *TERT* promoter was determined by Sanger sequencing, as described previously [43].

### 4.4. Next-Generation Sequencing

Libraries were diluted to a final concentration of 13 pM and sequenced using v3-600 cycles plates on a MiSeq^®^ sequencer (Illumina, San Diego, CA, USA). Raw sequences from samples with coverage of 300X in ≥70% of the regions were filtered and processed. SNV and indel variants with a variant allele frequency >5% were annotated using VariantStudio 3.0 (Illumina, San Diego, CA, USA) and Varsome [54] software (Saphetor, Boston, MA, USA). All pathogenic, likely pathogenic, and predicted pathogenic variants were visually checked with the Integrative Genome Viewer (IGV 2.3.32, Broad Institute, Cambridge, MA, USA).

### 4.5. Mechanistic Analysis of Pathways

To evaluate the functional implications of the individual mutational profiles, gene expression data of skin normal tissue were downloaded from GTEx data portal [55]. Using the normalized expression data from individuals, in silico knockdowns were simulated by multiplying the expression value by 0.01. Using KEGG signaling pathways topology information [56], each signaling pathway was decomposed in its functional circuits as described elsewhere [57], and the activation levels of each circuit were obtained for each mutational profile using tissue expression values after applying a re-scaling transformation of the rank of the matrix to (0, 1). An equal number of samples was randomly selected from GTEx skin tissue data to account for the different mutational profiles, obtaining a dataset of circuit activation levels from all samples, corresponding to each mutational profile.

Then, the differences in the circuits’ activation levels between the groups (CSD vs. nevogenic, CSD vs. normal tissue, and nevogenic vs. normal tissue) were evaluated. A linear model fit was performed and computed moderated *t*-statistics and log-odds of differential expression by empirical Bayes moderation using limma package from R/Bioconductor [58]. All *p*-values were adjusted for multiple comparisons using Benjamini and Hochberg FDR method. To account for random sample selection, a bootstrap of 50 iterations was performed and combined the statistical results using Fisher’s *p*-values combination method. We selected those circuits with an adjusted *p*-value < 0.05 and with a level of concordance of Fold Change values of 70% (meaning that at least 70% of the bootstraps showed a level of concordance in the sign of the fold change), obtaining a list of differentially activated circuits characteristic of each group (nevogenic and CSD).

These selected circuits were further annotated with the hallmarks of cancer using the Cancer Hallmarks Annotation Tool (CHAT) based in text-mining searching [59]. For each circuit, only those hallmarks with a score higher than the ninetieth percentile (0.18) were selected. To evaluate the impact over each hallmark of each group, the ratio of the number of significant circuits for each hallmark and the total of circuits annotated for each hallmark were calculated. Moreover, to evaluate the impact of the mutational profile over the whole pathway, a Fisher test was done to combine the individual values obtained from the independent circuits within the pathway in order to obtain the overall level of dysregulation of the whole signaling pathway.

A univariate enrichment analysis was ultimately performed to elucidate whether a hallmark was significantly enriched in each group with respect to normal skin. The *t*-statistic and the adjusted *p*-values obtained from both nevogenic vs. normal skin, and CSD vs. normal skin limma models were taken to obtain a ranking of the circuits together with the circuits annotated to hallmarks. Then, an analysis similar to a gene set enrichment analysis (gsea) was performed using Bioconductor msgsa R package to fit a logistic regression model relating the probability of circuits belonging to the functional hallmark set with the value of the ranking statistic.

### 4.6. Statistical Analysis

Clinical variables and mutational status for the analyzed genes were categorized. A chi-square test was applied to evaluate differences among the groups. Univariate and adjusted logistic regression models were used to establish the association between variables. A value of *p* < 0.05 was set to define significance. The statistical analyses were performed using IBM Corp. released 2011 (IBM SPSS Statistics for Macintosh, IBM Corp, version 20.0. Armonk, NY, USA).

## 5. Conclusions

We presented a detailed cohort of cutaneous melanoma patients classified into etiopathogenic groups showing distinct molecular profiles. These data provide further corroboration that the nevogenic and CSD melanoma subtypes, defined by the divergent pathway theory of melanoma, reflect the disease’s specific biology.

## Figures and Tables

**Table 1:** Demographic and clinicopathological characteristics of the cohort.

Variables		Total		Nevogenic		CSD	
		N	%	N	%	N	%
Sex	Male	65	54.6	42	51.2	23	62.2
	Female	54	45.4	40	48.8	14	37.8
Phototype	1	2	1.7	2	2.4	0	0
	2	32	26.9	23	28.0	9	24.3
	3	68	57.1	46	56.1	22	59.5
	4	15	12.6	10	12.2	5	13.5
	5	1	0.8	0	0	1	2.7
	Unknown	1	0.8	1	1.2	0	0
Sunburns at the area of melanoma	None	12	26.7	8	27.6	4	25.0
	Mild	15	33.3	9	31.0	6	37.5
	Severe	16	35.6	10	34.5	6	37.5
	N/A	1	2.2	1	3.4	0	0
	Unknown	1	2.2	1	3.4	0	0
Basal Cell Carcinoma	No	96	81.4	72	88.9	24	64.9
	Yes	22	18.6	9	11.1	13	35.1
Multiple Melanoma	No	109	93.2	76	93.8	33	91.7
	Yes	8	6.8	5	6.2	3	8.3
Familial Melanoma	No	102	87.2	67	82.7	35	94.6
	Yes	15	12.8	13	16.0	2	5.4
Anatomical location	Head/Neck	30	25.2	2	2.4	28	75.7
	Limb	25	21.0	18	22.0	7	18.9
	Trunk	59	49.6	57	69.5	2	5.4
	Acral	4	3.4	4	4.9	0	0
	Other	1	0.8	1	1.2	0	0
Histological type	LMM	18	15.1	1	1.2	17	45.9
	SSM	73	61.3	60	73.2	13	35.1
	NM	15	12.6	11	13.4	4	10.8
	ALM	3	2.5	3	3.7	0	0
	Desmoplastic	2	1.7	2	2.4	0	0
	Spitzoid	2	1.7	2	2.4	0	0
	Other	6	5.0	3	3.7	3	8.1
Ulceration	No	99	83.2	70	85.4	29	78.4
	Yes	20	16.8	12	14.6	8	21.6
Sentinel node	Negative	19	67.9	14	82.4	5	45.5
	Positive	6	21.4	3	17.6	3	27.3
	Unknown	3	10.7	0	0	3	27.3
Age *	<=59	59	50.0	58	71.6	1	2.7
	>59	59	50.0	23	28.4	36	97.3
Breslow *	<=1.08	59	50.0	48	59.3	11	29.7
	>1.08	59	50.0	33	40.7	26	70.3

* Categorized by the median of the studied population.

**Table 2:** Mutational prevalence in our cohort and classification into molecular subtypes.

Gene	Mutation Prevalence	Gene	Mutation Prevalence
*TERTp*	52.21	*RB1*	4.20
*BRAF*	50.42	*PIK3R1*	4.20
*NF1*	16.81	*GNA11*	4.20
*NRAS*	13.45	*CDK4*	3.36
*ROS1*	11.76	*PPP6C*	3.36
*TP53*	10.92	*PTEN*	2.52
*ARID2*	9.24	*HRAS*	1.68
*CDKN2A*	7.56	*MAP2K2*	1.68
*RAC1*	5.88	*GNAQ*	0.84
*IDH1*	5.04	*KRAS*	0.84
*KIT*	4.20	*PIK3CA*	0.84
Molecular subgroup	% within cohort	Molecular subgroup	% within cohort
“*BRAF+*”	40.3	“*BRAF+RAS+*”	2.5
“*RAS+*”	12.6	“*BRAF+NF1+*”	7.6
“*NF1+*”	8.4	“*RAS+NF1+*”	0.8
“3wt”	27.7		

**Table 3:** Prevalence of mutations according to etiopathogenic group and molecular subgroups.

Gene	Status	Total		Nevogenic		CSD		*p*-Value
		N	%	N	%	N	%	
*TP53*	WT	106	89.1	76	92.7	30	81.1	0.108
	Mutated	13	10.9	6	7.3	7	18.9	
*NF1*	WT	99	83.2	76	92.7	23	62.2	<0.001
	Mutated	20	16.8	6	7.3	14	37.8	
*BRAF*	WT	59	49.6	36	43.9	23	62.2	0.077
	Mutated	60	50.4	46	56.1	14	37.8	
*ROS1*	WT	105	88.2	78	95.1	27	73.0	0.001
	Mutated	14	11.8	4	4.9	10	27.0	
*NRAS*	WT	103	86.6	70	85.4	33	89.2	0.773
	Mutated	16	13.4	12	14.6	4	10.8	
*CDK4*	WT	115	96.6	79	96.3	36	97.3	1
	Mutated	4	3.4	3	3.7	1	2.7	
*ARID2*	WT	108	90.8	77	93.9	31	83.8	0.094
	Mutated	11	9.2	5	6.1	6	16.2	
*CDKN2A*	WT	110	92.4	76	92.7	34	91.9	1
	Mutated	9	7.6	6	7.3	3	8.1	
*KIT*	WT	114	95.8	80	97.6	34	91.9	0.173
	Mutated	5	4.2	2	2.4	3	8.2	
*RB1*	WT	114	95.8	79	96.3	35	94.6	0.646
	Mutated	5	4.2	3	3.7	2	5.4	
*PPP6C*	WT	115	96.6	79	96.3	36	97.3	1
	Mutated	4	3.4	3	3.7	1	2.7	
*PTEN*	WT	116	97.5	80	97.6	36	97.3	1
	Mutated	3	2.5	2	2.4	1	2.7	
*IDH1*	WT	113	95.0	78	95.1	35	94.6	1
	Mutated	6	5.0	4	4.9	2	5.4	
*GNA11*	WT	114	95.8	81	98.8	33	89.2	0.032
	Mutated	5	4.2	1	1.2	4	10.8	
*GNAQ*	WT	118	99.2	82	100.0	36	97.3	0.311
	Mutated	1	0.8	0	0	1	2.7	
*RAC1*	WT	112	94.1	81	98.8	31	83.8	0.004
	Mutated	7	5.9	1	1.2	6	16.2	
*KRAS*	WT	118	99.2	81	98.8	37	100.0	1
	Mutated	1	0.8	1	1.2	0	0	
*HRAS*	WT	117	98.3	81	98.8	36	97.3	0.527
	Mutated	2	1.7	1	1.2	1	2.7	
*MAP2K2*	WT	117	98.3	82	100.0	35	94.6	0.095
	Mutated	2	1.7	0	0	2	5.4	
*PIK3CA*	WT	118	99.2	82	100.0	36	97.3	0.311
	Mutated	1	0.8	0	0	1	2.7	
*PIK3R1*	WT	114	95.8	80	97.6	34	91.9	0.173
	Mutated	5	4.2	2	2.4	3	8.1	
*TERTp*	WT	54	47.8	41	51.9	13	38.2	0.220
	Mutated	59	52.2	38	48.1	21	61.8	
Pathogenic mutations	<=2	74	62.2	55	67.1	19	51.4	0.108
	>2	45	37.8	27	32.9	18	48.6	
Mutational subtype *	“*BRAF+*”	48	45.3	39	52.0	9	29.0	<0.001
	“*RAS+*”	15	14.2	12	16.0	3	9.7	
	“*NF1+*”	10	9.4	1	1.3	9	29.0	
	“3wt”	33	32.4	23	30.7	10	32.3	

* 13 tumors showing concurrent mutations from different subtypes were excluded.

## Data Availability

Data is available upon request to the corresponding author.

## Supplementary Materials

The following are available online at https://www.mdpi.com/article/10.3390/cancers13205219/s1, Figure S1: Sample flowchart, Figure S2: Molecular subtype distribution, Figure S3: Mutation heatmap, Figure S4: Genes OR to be associated with CSD, Figure S5: Heatmap of dysregulated circuits, Figure S6: Enrichment analysis, Figure S7: Genes included in the amplicon panel.
