# Supplementary material for: Mutational Characterization of Cutaneous Melanoma Supports Divergent Pathways Model for Melanoma Development
Source: Cancers (Basel). 2021 Oct 18;13(20):5219. doi: 10.3390/cancers13205219 (PMC8533762; doi:10.3390/cancers13205219)
Supplement: Supplementary file 1 [file cancers-13-05219-s001.zip › cancers-1373348-supplementary.pdf]

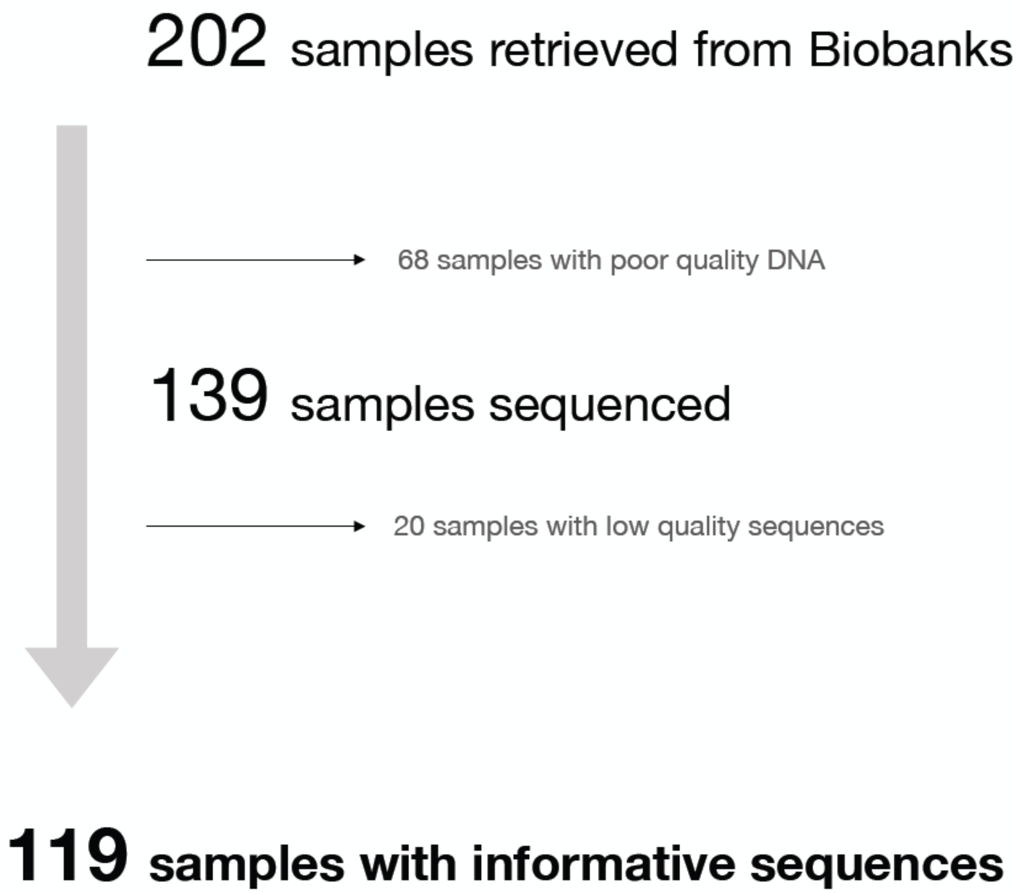

Figure S1. Sample flowchart. Here we can see the evolution in the number of samples after each step in the process.

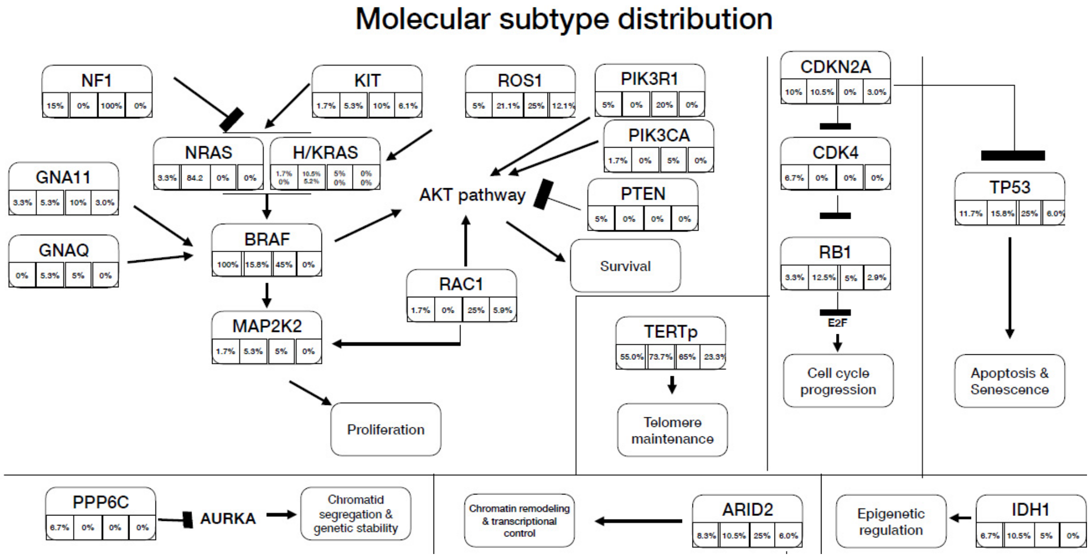

Figure S2. Molecular subtype distribution. Here we see the mutational prevalence of each gene for the non-exclusive four molecular subtypes BRAF+, RAS+, NF1+, 3wt. The lines indicate the downstream direction in each pathway: a normal arrow means activation, while a bar means

inactivation

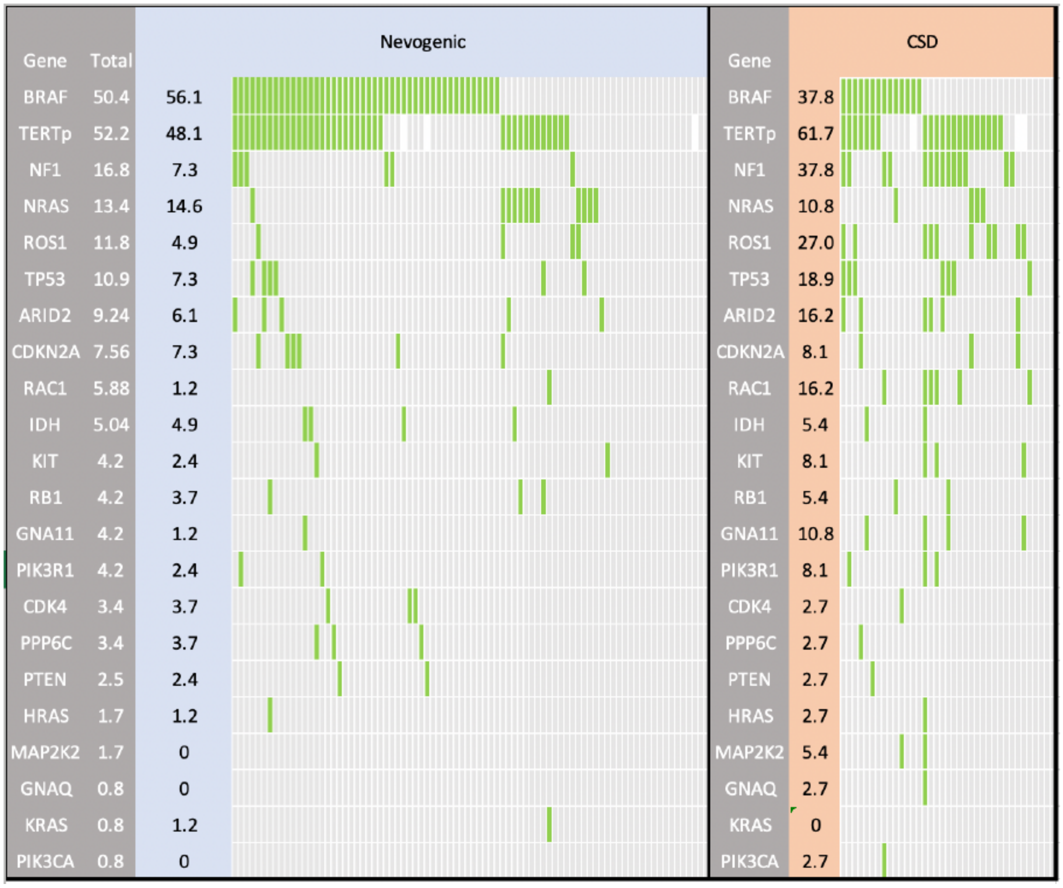

Figure S3. Mutation Heatmap. Graphical description of mutational distribution among the different genes for both groups, together with the total and group prevalence in percentage. Each column represents one sample. Cells are colored green when mutated, grey when wild-type, and white when non-informative.

Variable	Univariate				Multivariate			
	OR	95% CI		p value	OR	95% CI		p value
		Lower	Upper			Lower	Upper	
TP53+	3.0	0.9	9.5	0.069	-	-	-	-
NF1+	7.7	2.7	22.3	<0.001	5.654	1.765	18.118	0.004
BRAF+	0.5	0.2	1.1	0.067	-	-	-	-
ROS1+	7.2	2.1	24.9	0.002	5.658	1.433	22.346	0.013
NRAS+	0.7	0.2	2.4	0.573	-	-	-	-
CDK4+	0.7	0.1	7.3	0.790	-	-	-	-
ARID2+	3.0	0.8	10.5	0.089	-	-	-	-
CDKN2A+	1.1	0.3	4.7	0.880	-	-	-	-
KIT+	3.5	0.6	22.1	0.178	-	-	-	-
RB1+	1.5	0.2	9.4	0.662	-	-	-	-
PPP6C+	0.7	0.1	7.3	0.790	-	-	-	-
PTEN+	1.1	0.1	12.7	0.932	-	-	-	-
IDH1+	1.1	0.2	6.4	0.903	-	-	-	-
GNA11+	9.8	1.1	91.2	0.045	7.097	0.615	81.911	0.116
GNAQ+	-	-	-	-	-	-	-	-
RAC1+	15.7	1.8	135.5	0.012	7.519	0.673	83.950	0.101
KRAS+	-	-	-	-	-	-	-	-
HRAS+	2.3	0.1	37.0	0.570	-	-	-	-
MAP2K2+	-	-	-	-	-	-	-	-
PIK3CA+	-	-	-	-	-	-	-	-
PIK3R1+	3.5	0.6	22.1	0.178	-	-	-	-
TERTp+	1.7	0.8	4.0	0.184	-	-	-	-

Figure S4. Genes OR to be associated with CSD (Nevogenic is reference).

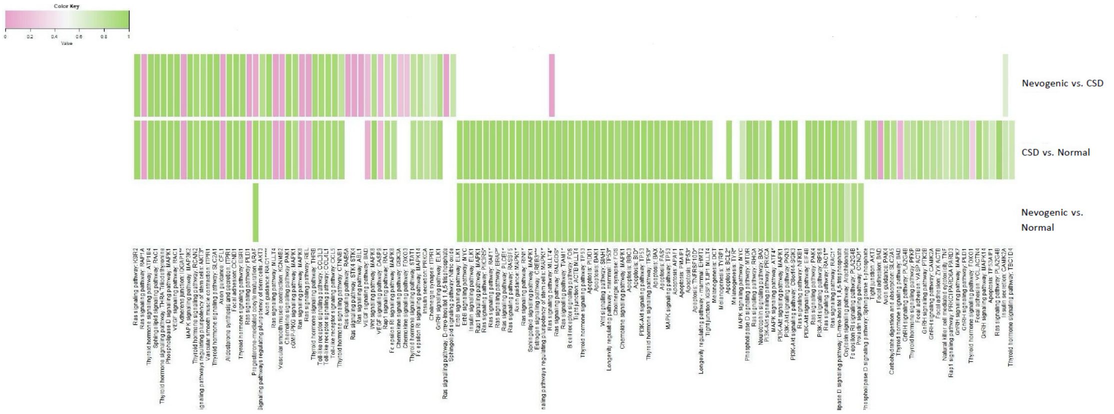

Figure S5. Heatmap of dysregulated circuits. This graphical representation shows with a color key whether certain circuits are infra-expressed (pink) or over-expressed (green) with respect to their reference. The lower row compares nevogenic melanomas vs. normal skin tissue; the middle one compares CSD melanomas vs. normal skin tissue; and the upper one compares nevogenic melanomas vs. CSD melanomas. We can see how CSD melanomas have more dysregulated circuits, which are mostly overexpressed.

Cancer Hallmark	p value	
	Nevogenic	CSD
Cellular Energetics	0.1795	0.1720
Evading Growth Suppressors	0.1353	0.2803
Genome Instability and Mutation	0.2756	0.4863
Immune Destruction	0.6243	0.5718
Inducing Angiogenesis	0.2487	0.4201
Invasion and Metastatsis	0.5365	0.8989
Replicative Immortality	0.0026	0.0002
Resisting Cell Death	0.3328	0.4693
Sustaining Proliferative Signaling	0.0103	0.0004
Tumor Promoting Inflammation	0.5929	0.2058

Figure S6. Enrichment analysis table. Here we can see for both groups whether a hallmark is statistically significantly enriched in melanoma with respect to normal skin.

Gene	Chromosome	Location GRCh37	Number of target regions	Number of target bases	Number of bases covered	% bases covered	Number of amplicons	Size minimum	Size average	Size maximum
ARID2	chr12	46123609	21	5,928	5,928	100.0	57	157	208	225
BRAF	chr7	140415817	20	2,86	2,846	99.5	36	130	199	225
CDK4	chr12	58142297	7	1,052	1,052	100.0	12	131	186	225
CDKN2A	chr9	21968217	6	1,184	892	75.3	11	120	185	244
GNA11	chr19	3094639	7	1,22	1,064	87.2	14	156	202	234
GNAQ	chr9	80336228	7	1,22	1,064	87.2	13	188	214	225
HRAS	chr11	532578	5	780	780	100.0	11	129	193	255
IDH1	chr2	209101792	8	1,405	1,405	100.0	18	145	200	225
KIT	chr4	55524171	21	3,354	3,354	100.0	41	142	200	225
KRAS	chr12	25362718	5	787	787	100.0	11	130	180	224
MAP2K2	chr19	4090585	11	1,423	1,242	87.3	19	135	192	275
NF1	chr17	29422317	60	9,9	9,9	100.0	115	130	204	240
NRAS	chr1	115251145	4	650	650	100.0	7	174	207	224
PIK3CA	chr3	178916603	20	3,607	3,546	98.3	46	147	204	264
PIK3R1	chr5	67522493	17	2,637	2,637	100.0	28	130	201	225
PPP6C	chr9	127911941	8	1,189	1,189	100.0	14	134	191	225
PTEN	chr10	89624216	9	1,392	1,375	98.8	17	141	196	237
RAC1	chr7	6414356	7	776	721	92.9	10	130	186	225
RB1	chr13	48878038	27	3,327	3,307	99.4	45	130	200	268
ROS1	chr6	117609644	43	7,904	7,904	100.0	88	136	203	225
TP53	chr17	7572916	12	1,503	1,503	100.0	20	130	180	225
**TOTAL**			325	54,098	53,146	98.2	633	120	200	275

Figure S7. Genes included in the amplicon panel. Here we have the details for the gene panel.
